# Reducing greenhouse gas emissions and improving air quality: Two global challenges

**DOI:** 10.1002/ep.12665

**Published:** 2017-06-29

**Authors:** Larry E. Erickson

**Affiliations:** ^1^ Department of Chemical Engineering Kansas State University, Durland Hall Manhattan Kansas 66506

**Keywords:** solar, wind, electric vehicles, charging infrastructure, smart grid, climate change

## Abstract

There are many good reasons to promote sustainable development and reduce greenhouse gas emissions and other combustion emissions. The air quality in many urban environments is causing many premature deaths because of asthma, cardiovascular disease, chronic obstructive pulmonary disease, lung cancer, and dementia associated with combustion emissions. The global social cost of air pollution is at least $3 trillion/year; particulates, nitrogen oxides and ozone associated with combustion emissions are very costly pollutants. Better air quality in urban environments is one of the reasons for countries to work together to reduce greenhouse gas emissions through the Paris Agreement on Climate Change. There are many potential benefits associated with limiting climate change. In the recent past, the concentrations of greenhouse gases in the atmosphere have been increasing and the number of weather and climate disasters with costs over $1 billion has been increasing. The average global temperature set new record highs in 2014, 2015, and 2016. To reduce greenhouse gas emissions, the transition to electric vehicles and electricity generation using renewable energy must take place in accord with the goals of the Paris Agreement on Climate Change. This work reviews progress and identifies some of the health benefits associated with reducing combustion emissions. © 2017 American Institute of Chemical Engineers Environ Prog, 36: 982–988, 2017

## INTRODUCTION

There is significant recent progress in reducing greenhouse gas emissions and considerable new information has appeared in the published literature 1, 2, 3, 4, 5, 6, 7, 8, 9, 10, 11, 12, 13, 14, 15, 16, 17, 18, 19, 20, 21, 22, 23, 24, 25, 26, 27, 28, 29, 30, 31, 32, 33, 34, 35, 36, 37, 38, 39, 40, 41, 42, 43, 44, 45, 46, 47, 48, 49, 50, 51, 52, 53, 54, 55, 56, 57, 58, 59, 60, 61, 62, 63, 64, 65, 66, 67, 68, 69, 70, 71, 72, 73, 74, 75, 76, 77, 78, 79, 80, 81, 82, 83, 84, 85, 86, 87, 88, 89, 90, 91, 92, 93, 94, 95. This work reviews recent research and commercial progress related to the electrification of transportation and the transition to sustainable energy. One of the significant accomplishments by the United Nations and the partner countries was the approval of the Paris Agreement on Climate Change on December 12, 2015 at the United Nations Framework Convention on Climate Change 80. The task of reducing greenhouse gas (GHG) emissions such that their concentrations in the atmosphere stop increasing is a very significant global challenge. To accomplish this goal, it is necessary to electrify transportation, generate electricity without carbon emissions, electrify agricultural operations, heat buildings with solar energy and electricity, and reduce carbon emissions associated with construction, mining, and industrial production. One important benefit of doing all this is better air quality because of reduced emissions. Society values health and environmental qualities such as clean air and ambient temperatures that are comfortable for outdoor activities.

The transition to a carbon neutral world is a great challenge, and it will take many years. The goal to reduce GHG emissions by 80% by 2050 is considered to be reasonable 32, 85. The state of California has a goal to reduce GHG emissions by 40% compared with 1990 by 2030 19. The German government has set a goal of 40% reduction relative to 1990 by 2020.

There is some recent progress with respect to the goal of reducing emissions 2, 5, 39, 64. Between 2000 and 2014, 21 countries reported increases in Gross Domestic Product (GDP) while reducing GHG emissions 2. For example, Denmark had a 30% reduction in GHG emissions and 8% increase in GDP while Germany had 12% reduction in GHG emissions and 16% increase in GDP. The state of California is making good progress on its effort to reduce greenhouse gas emissions and improve air quality 21. A number of books related to the topics of this review have been published recently 15, 32, 42, 74.

There are many impacts related to greenhouse gas emissions and the increase of their concentrations in the atmosphere 30, 42, 74. Global average temperatures have been increasing and a new record was set in 2016 33, 60. This is the third year in a row in which a new global average temperature was set, and the average global temperature across land and ocean was 0.94 degrees Celsius above the 20th century average temperature 60.

This review will address issues related to the electrification of transportation, the transition to the generation of electrical power with renewable energy, the electrification of agriculture, and research and development needs to accomplish this transition.

## PARIS AGREEMENT ON CLIMATE CHANGE

The Paris Agreement on Climate Change is voluntary. Each country has the freedom to move forward to reduce its greenhouse gas emissions along a pathway that works for it. The agreement went into force on November 4, 2016 because more than 55 countries that emit more than 55% of the greenhouse gases had ratified the agreement as of October 5, 2016, when a 30 day waiting period started. As of November 3, 2016, 94 countries had signed the agreement and ratified it, representing 66% of global emissions 13. Obama 64 has reported that more than 110 countries representing more than 75% of global emissions have ratified the agreement. One goal of the agreement is to reduce greenhouse gas emissions sufficiently to keep global warming to <2°C. Another goal is to reduce greenhouse gas emissions sufficiently to approach a steady state condition in which the concentrations of greenhouse gases in the atmosphere are approximately constant or decreasing by 2100 [80]. This occurs when the emissions are approximately equal to the removal of the gases from the atmosphere. The removal of carbon dioxide from the atmosphere by photosynthetic processes in water and land is significant; however, the carbon incorporated into organic molecules needs to be sequestered to help with the carbon balance.

Future impacts of climate change will be reduced by keeping the increase in temperatures to 2°C or less. Climate change impacts agriculture, flooding is a greater problem, sea level rise causes coastal problems, there are health and economic impacts, and human migration has increased 11, 30, 42, 74. Burke *et al*. 16 have estimated that if no action is taken to reduce greenhouse gas emissions, in 2100 economic production will be 23% less than if there was no climate change 30. Obama 64 has reported estimates of $340 billion to $690 billion in lost revenue annually if the average global temperature rises by 4°C. Air quality will be impacted by wild fires and dust from drought conditions that are made worse by climate change 4.

One of the benefits associated with reducing greenhouse gas emissions is an improvement in air quality. Air quality is improved by electrifying transportation because emissions from vehicles are a significant part of smog and urban air pollution 30, 32, 38. The particulates in diesel exhaust may cause cancer 1, 32, 41, 86. The World Health Organization Air Quality Guideline for particulates is 10 μg/m^3^ in air, and many cities of 1 million or more people do not meet this guideline 14, 87. Particulate matter in air has been found to be the sixth largest overall risk factor for global premature mortality 6, 30. There are many premature deaths associated with asthma, chronic obstructive pulmonary disease, and cardiovascular disease. Having diabetes increase the chances of harm from air pollution 4. Lung cancer risk is higher for those exposed to diesel particulates 1, 79.

The most important contaminants in outdoor air are particulates in most environments, and a significant portion of the particulates are from vehicle engine combustion 6, 14, 30, 32. The concentrations of particulates along streets and roads vary with time and traffic at a fixed location 10, 12 and they vary with position because of traffic and buildings. Concentrations of particulates are often larger near streets and roads compared to those at ambient air quality monitoring stations 4, 12. Health effects associated with living near busy streets and roads include increased risk of Alzheimer's disease and dementia 17, 18, 23, 65, 79.

One way to reduce air pollution in major cities is to limit travel in the inner city to electric powered vehicles during certain periods of time. This option may be implemented by making the regulation effective at a future date. London, England has daily fees, with larger fees for older diesel vehicles, to reduce congestion and emissions in central London 94.

Coal fired power plants and furnaces are important sources of particulates also. Replacing coal combustion with renewable energy reduces greenhouse gas emissions and it improves air quality. The concentration of mercury in the atmosphere has been decreasing because of reduced mercury emissions from coal fired power plants 77. There are significant economic benefits associated with reducing mercury and carbon dioxide emissions from coal fired power plants 16, 64, 77.

In 2016, a record 14,625 megawatts of new solar power generation was installed in the United States. This is a 95% increase compared to 2015, and accounted for 39% of all new electric generating capacity added in the U.S. in 2016. For the first time, new solar power generation was ranked number one compared to all sources of power added in 2016 [59]. The largest fraction of this new generation was added by electric utilities.

## ELECTRIFICATION OF TRANSPORTATION

One of the changes that has started in the world is the electrification of transportation. As of 2017 there are more about 2 million electric vehicles in service 21. As shown in Table 1, the number of models for sale in the United States, U.S. sales, and global sales have been increasing rapidly 91, 92. In Norway more than 25% of new car sales are electric vehicles 7. In California, there are 30 cities in which the fraction of new car sales that are plug‐in vehicles ranges from 6 to 18% 8. There are incentives in California to encourage electric vehicle ownership because of the importance of better air quality 19. More than 40% of electric vehicle sales in the USA are in California 84. In a recent survey, those with electric vehicles liked the low cost of operation. The limited range was the most significant concern 21.

**Table 1:** Plug‐in electric auto sales.

Year	Number of models	USA	World
2012	9	52,607	125,760
2013	16	97,507	212,986
2014	22	122,507	320,713
2015	27	116,099	550,297
2016	31	158,614	777,497

Source: Data from Inside EVs 91, 92. Number of models is for sales in USA.

The transition to electric vehicles has many aspects to it and trillions of dollars will be expended. Developing a charging infrastructure that is necessary to support the electrification of transportation is one of the important challenges 32, 62, 89. The global opportunities associated with electric vehicles are still being identified and developed. Inexpensive simple automobiles and buses are possible for use in developing countries where people with limited incomes need transportation. Progress in batteries is very important because they are a major cost associated with new car sales.

There has been significant progress in the development of batteries for electric vehicles 32, 63. The cost of batteries has decreased substantially and there has been some technological progress 51, 72. Because of these developments, the range of new cars has increased compared to earlier models and prices of new electric vehicles have decreased, also. One of the important developments is the new battery factory in Nevada, which will reduce the cost of batteries to about $130/kWh 67. This means that the cost of 70 kWh of batteries for an electric vehicle will cost about $9100. There has been about a 7% annual increase in energy density as a result of innovation that has allowed for an increase in the range of electric vehicles 51. Edelstein 28 reports that the battery cell cost is about $145/kWh in the new Chevy Bolt for a 60 kWh battery pack that has a range of 238 miles. He also reports estimates of battery cell prices of $100/kWh by 2020 and $80/kWh after 2020.

There is significant research activity related to new batteries 22, 32, 58. Solid state batteries that do not have liquid electrolytes have been developed and they have the potential to increase energy density and reduce cost 22, 58. The U.S. Department of Energy supports battery materials research, applied research and advanced battery development with the goals to (1) Reduce production cost; (2) Decrease the size of electric batteries; and (3) Decrease the weight of electric vehicle batteries 82. In their literature review, Wolfram and Lutsey 88 point out that new batteries with significantly greater energy and power densities are being developed.

The charging infrastructure for electric vehicles is developing, and it is now possible to travel long distances in battery electric vehicles with a range of more than 200 miles (322 km). Electric vehicle charging includes level 1, level 2, and fast charging 32. In some cases, the electric vehicle supply equipment (EVSE) is covered by solar panels or inside buildings. Shade is very beneficial in warm and hot climates because battery life is much longer if batteries do not overheat 9, 32, 56. Thus, when charging batteries in a hot climate, shade should be provided; this can be accomplished by covering charging stations with solar panels 31, 32, 34, 71.

Tesla has developed a network of fast charging stations along major roads in many parts of the world 32, 78. While initially, charging was free for Tesla owners, in 2017, the company began to change this policy 78. The installation of EVSE equipment to allow fast charging along major highways, workplace charging, and charging at home are the three most important infrastructure needs 32.

There is a significant effort to add solar powered charging infrastructure in China and to install fast charging along some major highways 26, 37, 89. Incentives to purchase electric vehicles 55 have increased electric vehicle sales in China to more than 40,000 new vehicles/month 68. In November 2016, the 43,441 sales of new electric vehicles in China was the most of any country and China has moved ahead in number of electric vehicles in the country with more than 650,000 in service 68.

Adding solar powered charging stations at many locations is helpful in addressing the concern of consumers about the range of electric vehicles. The addition of charging infrastructure has significant value to electric utilities because it allows for charging at more places and more times of the day. If electric companies have time of use or real time prices, there are more opportunities to charge when it is beneficial to the task of balancing supply and demand. As the solar fraction of power generation increases, workplace charging with solar power generated at the parking lot may be very cost effective.

One of the newer developments is wireless charging where the electric vehicle is parked over a pad, and the pad on the ground sends electric power to the pad on the car 70. Mercedes‐Benz and Qualcom are working cooperatively on this development. The Mercedes S550e plug‐in hybrid sedan is scheduled to have this option in 2018 [70]. The convenience of arriving home or at work and only needing to park in a specific location has value; however, there is still the need to manage the charging from the standpoint of real time pricing of electricity. There is an effort to work with SAE International to set a global wireless electric vehicle charging standard 70.

Volta is a company that installs EVSE in shopping center and retail store parking lots and provides free charging to customers. The cost of the installations is paid for by advertising at the EVSE 32. The free charging increases business at the locations where they are available.

As the cost of energy storage decreases, there is a greater emphasis on including energy storage at locations where charging is available. Many of the sites with fast charging have energy storage because of demand charges of electric power companies and the magnitude of the power flow to the electric vehicle that is being charged 32, 76. EVgo has broken ground on a 350kW fast charging station with energy storage and solar panels to generate some of the electric power 76. Fast charging ranges from about 30 to 50 kW historically, but this new station will have the ability to go up to 350 kW. One of the desires of travelers is to recharge quickly when out on a long trip 76. At a charge rate of 100 kW, it takes 30 min to add 50 kWh to the battery pack, which is sufficient to travel about another 150 miles (241 km). At 300 kW, the time to add 50 kWh is only 10 min. The cost of fast charging has been reported to be $0.22/kwh in Sacramento, California, at a solar panel covered parking lot operated by the Sacramento Municipal Utility District 30.

Another application for energy storage is in parking lots with solar powered charging stations where there are charging demands that are associated with events such as a football game or an evening event. As the number of electric vehicles in service increases, there will be greater demand for charging while at an event or meeting, and energy storage is helpful in meeting customer expectations.

One of the newer developments that has the potential to be of significant value to society is that of electric buses. There are now 4 companies, BYD, New Flyer Industries, Proterra and Volvo that are manufacturing electric buses 30. Wireless charging has the potential to be of significant value in bus charging because buses can stop to pick up passengers and the charging can be operational while the bus is stopped. If all city buses were electric buses, this would reduce greenhouse gas emissions and improve air quality. The Chicago Transit Authority estimates a savings of $80,000 per year by switching to electric buses ($25,000 in fuel and $55,000 in public health costs because of better air quality) 27, 30. Some individuals think that electric buses will capture the city transit bus market by 2030 [29].

## ELECTRICAL GRID AND RENEWABLE ENERGY

The last 10 years have been a time of change for electric power companies with significant growth in power generated with wind and solar energy. Brown *et al*. 15 have a book on this great transition to wind and solar power. Coal fired power plants are being replaced by natural gas, wind, and solar energy. Obama 64 reports a 41% decrease in electric power costs for wind generated electricity from 2008 to 2015, and a decrease of 54% in costs for roof top solar photovoltaic generated power. In recent reports, the amount of new wind capacity added is number one and the quantity of new solar capacity added is number two in 2015 30. This growth of wind and solar generating capacity is one of the reasons that greenhouse gas emissions have been decreasing 64.

One of the important facts associated with wind and solar generated power is that the amount of power generated at a particular time depends on local conditions. This requires a greater effort to manage demand by having real time prices and time of use prices for electricity. The smart grid with good communication with customers on prices for electricity can have benefits because customers can shift some of their uses to low price times and help balance supply and demand 32. Significant advances in new smart grid technology are being implemented by electric utilities 81. In order to have time of use prices, utilities need to have meters at each customer location that are able to record the amount of electricity that is used at each price 20. When time of use prices were made available to all customers, there was a reduction of electricity use during peak power intervals 20.

Energy storage that can be used to help manage demand becomes more important as the fraction of electricity generated with wind and solar increases. The decrease in the cost of Li‐ion batteries has made energy storage more cost effective 32. It has economic value when applied to the management of electricity demands over the 24 h cycle. The global battery storage market in 2015 was about 1.4 GWh (1.4 million kWh) 93. Batteries are not cost effective to manage seasonal differences in electricity demand.

The large storage capacity of the battery packs in newer electric vehicles has the potential to be of significant value in managing electrical power supply and demand. Batteries can be charged at times when there is a need to use more electricity to balance supply and demand. Prices need to be communicated and there needs to be sufficient incentives to have a good response.

## ELECTRIFICATION OF AGRICULTURE

In December 2016, John Deere announced that they had an electric tractor under development 47, 49. The tractor is SESAM (Sustainable Energy Supply for Agricultural Machinery) and it has two 150 kW electric motors and 130 kWh lithium ion battery pack. The two motors are designed so one of them can be used for farming operations while the other moves the tractor. Janzen 44 points out that farmers are more likely to begin using electric pickup trucks and small electric utility tractors because the range issue is not a problem in most applications and farms have space for solar panels on the roof of a building or another location. The Kharkov tractor plant has started to sell electric tractors in Ukraine 57. The Swedish Institute of Agricultural and Environmental Engineering has been investigating electric tractors and their applications 35. The Valley Oak Tool Company has converted Farmall Cub tractors into electric tractors 83. Some research on combining solar generated electricity with electric tractors has been reported 3. Work on standards for the electrification of tractors and various implements has started 24.

Progress to electrify agriculture is far behind the electrification of transportation in the United States and in the world. Progress with respect to the installation of solar panels and wind energy for agriculture is modest. Electricity that is generated on farms may be connected to the electrical grid and/or battery storage to manage the power that is produced and have power when it is needed.

## ECONOMICS

Because of scientific and technological progress, prices have been falling for batteries and solar panels. The cost of electric vehicles has been decreasing and vehicle range has been increasing because of progress to increase battery density and reduce battery cost. Costs have also decreased because of experience and competition. The cost of solar powered charging infrastructure has decreased as solar panel costs decreased and solar panel efficiency increased 32.

If all costs are considered, the following are already cost effective and commercially viable in many locations:

1. Wind generated electricity
2. Solar generated electricity
3. Electric vehicles
4. Solar powered charging stations
5. Battery storage

There are two important economic reasons to move forward with advances in renewable energy and the electrification of transportation. These are the costs of climate change and air pollution. Table 2 shows the costs associated with weather and climate disasters in the United States from 2000 to 2016 for events that have costs of $1.0 billion or more. The number of these events has been increasing. Hurricane Katrina at $125 billion in 2005 and hurricane Sandy at $65 billion in 2012 are the two most costly events 61. The drought in 2012 had a cost of $30 billion and impacted 1,584 counties in 32 states including Colorado, Indiana, Kansas, Nebraska, and Oklahoma 61, 95. The flooding in Louisiana in August 2016 cost $10 billion 61.

**Table 2:** Number of weather and climate disasters and cost each year from 2000 to 2016, in billions of dollars, in the United States 61.

Year	Number of events	Wildfires	Drought	Severe weather	Hurricanes
2000	2	1.1	5.0		
2001	2			11.6	
2002	4	1.1	9.0	2.1	1.1
2003	7	3.9	5.0	8.2	5.5
2004	5			1.0	54.3
2005	5		1.5		165.0
2006	6	1.5	6.0	6.8	
2007	5	2.7	3.5	5.9	
2008	11	1.2	7.0	18.7	37.3
2009	7	1.0	3.5	7.0	
2010	5			7.3	
2011	16	1.4	12.0	40.1	13.5
2012	11	1.7	30.0	16.3	67.8
2013	9		10.4	12.3	
2014	8		4.0	13.2	
2015	10	3.0	4.5	14.9	
2016	15	2.0	3.5	30.5	10.0

The global social cost of air pollution is at least $3 trillion per year 30, 32, 46, 53. There are health care costs and income loss due to being unable to work associated with cardiovascular disease, chronic obstructive pulmonary disease, asthma, and lung cancer. Premature mortality is included in some cost estimates also 6. The worldwide cost of dementia is over $800 billion/year 69. The American Lung Association of California report 38 includes costs of $24 billion for health and $13 billion for climate that are attributed to passenger vehicle air pollution for the states of California, Connecticut, Maine, Massachusetts, New Jersey, New York, Rhode Island, and Vermont.

The economic incentives to electrify transportation are very great when the costs associated with air pollution and climate change are considered. The cost of air pollution are being considered in California, and this is one of the reasons for the incentives that are provided to those who purchase electric vehicles 21.

## RESEARCH NEEDS

There is a continuing research effort and many new developments related to the transitions to renewable energy and electrification of transportation. Research on developing the smart grid and charging infrastructure to support these efforts is ongoing in many countries 25, 32, 48, 50, 52, 54, 75. There is ongoing research to reduce the cost of production and improve the efficiency of solar panels for electricity generation 66, 90. New materials such as perovskite have the potential to produce solar panels with higher efficiency 66, 73, 90. Perovskite crystals on silicon is being investigated also. Battery research to reduce cost, increase energy density, and improve the life time remains a high priority.

The development of electric vehicle charging infrastructure and methods for charging are progressing with active research on the rate of charging, wireless charging, and charging while driving 25, 52, 75. It may be possible to embed charging infrastructure in roads such that vehicles can drive over the wireless charger with a vehicle that can charge as it drives on the road because it has wireless charging capability 75.

Systems research is needed to develop optimal charging infrastructure and operational systems that serve society effectively. This includes integration of the smart grid, charging infrastructure, time of use pricing of electricity, and battery storage to have an effective operational system. The pricing of electricity with solar panels on homes, businesses, and in parking lots must be accomplished with fairness and appropriate incentives. There are research issues of vehicle charging associated with large events that draw 50,000 people or more. Optimal investment in charging infrastructure has started, but more work is needed 45.

There is some research on integration of solar panels into buildings with products such as solar shingles for homes and commercial buildings. Solar shingles are available, but more research is needed to reduce cost, improve efficiency of conversion of solar radiation to electricity, and increase the number of years of life of the product 36. Window awnings with solar panels to generate electricity are under development also.

## CONCLUSIONS

Reducing greenhouse gas emissions sufficiently to stabilize greenhouse gas concentrations in the atmosphere is a very important global challenge. Electrifying transportation and transitioning to renewable power sources for electricity are two very important goals that must be included in the effort. There has been great progress in reducing the cost of electrical power from renewable sources and in the development of electrical vehicles including cars and buses. Battery prices have reached the point where electric vehicles are competitive. There are now more than 2 million electric vehicles in service and new solar power generation in the US increased to 14,625 megawatts in 2016, a 95% increase compared to 2015.

There are social, environmental and economic reasons to move forward with programs and efforts to reduce combustion emissions. Health impacts of vehicle emissions are significant and costly. Both air quality and climate change impacts are significant; however, they are present as economic externalities. Policy action is needed to encourage progress. The Paris Agreement on Climate Change is important and we should all work together to make it successful.
